# Preparation and Interfacial Properties of Hydroxyl-Containing Polyimide Fibers

**DOI:** 10.3390/polym15041032

**Published:** 2023-02-19

**Authors:** Jiang Du, Chuanzhi Pu, Xianyu Sun, Qi Wang, Hongqing Niu, Dezhen Wu

**Affiliations:** State Key Laboratory of Chemical Resource Engineering, School of Materials Science and Engineering, Beijing University of Chemical Technology, Beijing 100029, China

**Keywords:** polyimide fibers, hydrogen bonding, mechanical properties, composites, interfacial shear strength

## Abstract

Developing polyimide (PI) fibers with excellent interfacial adhesion and high mechanical properties for the PI fiber-reinforced polymer matrix composites (PFRPs) industry has been challenging. In this work, 4,4′-diamino-(1,1′-biphenyl)-3,3′-diol (HAB) diamine was introduced into the rigid molecular chains, and the high-performance PI fibers, presenting an interfacial shear strength (IFSS) value of 46.33 MPa, tensile strength of 2.62 GPa, and modulus of 100.15 GPa, were successfully manufactured when the content of HAB in mixed diamines was 30 mol %. Fourier transform infrared (FTIR) spectroscopy identified the presence of intermolecular H-bonding interactions, and 2D small-angle X-ray scattering indicated that the introduction of HAB moiety contributed to reducing the radii of microvoids in the fibers, which were considered to be the key factors leading to a significant enhancement in the mechanical properties of the fibers. X-ray photoelectron spectroscopy (XPS) and the static contact angle intuitively illustrated that the synthetic fiber surface contained active hydroxyl groups. The IFSS value of PI fiber/epoxy resin composites (PI/EPs) was 56.47 MPa when the content of HAB reached 70 mol %. Failure morphologies confirmed that the interfacial adhesion of PI/EPs was enhanced owing to the surface activity of PI fibers. Consequently, this study provides an effective strategy to the long-standing problems of high mechanical performances and poor surface activity for traditional PI fibers used in the PFRPs industry.

## 1. Introduction

For nearly a century, many efforts have been concentrated on developing high-performance synthetic fibers for hi-tech industrial applications, due to the outstanding characteristics of fiber-reinforced polymer matrix composites (FRPs), such as low weight, high ratio of tensile strength to weight, excellent stiffness, and versatile designability [1]. For example, carbon fibers (CFs) have been maturely applied to the composites of aircraft and automotive fields; ultra-high molecular weight polyethylene (UHMWPE) fibers with outstanding specific absorption energy are widely utilized as reinforcement in bulletproof and stab-proof composites; and poly-p-phenylene benzobisoxazole (PBO) fibers with excellent mechanical–thermal properties have become an ideal reinforcement for composites in aerospace, military, and transportation fields [2,3,4]. Unfortunately, these facts, including the brittleness of CFs [5], the low glass transition temperature of UHMWPE [6], and the non-UV resistance of PBO [7], limit the use performance, application fields, and service life of such composites to some extent. Therefore, searching for high-performance fibers with excellent comprehensive properties as composite reinforcement has become one of the current research hotspots.

Over the past few decades, owing to the continuous innovations in spinning solution synthesis technology and spinning equipment, aromatic polyimide (PI) fibers with high mechanical properties, outstanding irradiation resistance, excellent thermal stability, high toughness, good chemical resistance, and low densities have not only attracted the close attention of researchers [8,9,10], but also have achieved satisfactory application effects in the composites industry. For instance, He et al. [11] successfully developed the hybrid fiber-reinforced polymer (HFRP) with high strength and excellent toughness by using CF (T700) and PI fibers (S35) as reinforcements. Zhang et al. [12] fabricated flexible Ni-Fe-P/PPy@PI fiber paper-based composites for 5G electromagnetic shielding fields. Gu et al. [13] prepared the PI/bismaleimide resin composites with anti-highspeed impact properties via the autoclave molding process, etc. However, the interfacial adhesion for advanced composites directly determines the effect of the resin matrix transferring external loads to the reinforcement with reinforcing and toughening roles [14]. Generally, the backbones of commercial PI fibers are mainly composed of benzene rings and cyclic imides, which makes its surface chemically inert and thus leads to poor interfacial adhesion between the fiber and resin matrix. For this reason, improving the interfacial adhesion has become an urgent demand for the preparation of high-performance PI fiber-reinforced polymer matrix composites (PFRPs).

Up to now, to enhance the interfacial adhesion between PI fibers and resin, academia and industry have made great efforts to develop a variety of methods to modify the fiber′s surface, such as alkali solution treatment, plasma, surface chemical grafting, and surface coating [15,16,17,18,19]. In detail, Zhou et al. [20] used a silane-coupling agent (3-aminopropyltriethoxysilane, KH-550) to modify the surface of PI fibers; when the concentration of KH-550 was 4 wt %, the interfacial shear strength (IFSS) value between fiber and matrix increased to 46.2 MPa. Lu et al. [16] proposed an oxygen plasma–silane coupling agent method to treat the surface of PI fibers. The results showed that after plasma treatment for 27 min, the IFSS between PI fibers coated with the silane coupling agent and matrix reached the highest value of 38.82 MPa. Zhang et al. [19] designed novel short-cut PI fibers modified by multi-walled carbon nanotubes (MWCNTs). Compared with the pure polycarbonate/acrylonitrile-butadiene-styrene copolymer (PC/ABS) alloy, when the fiber content was 20 wt %, the notched impact, tensile, and bending strength of MWCNT-decorated PI/ABS composites increased by 95%, 164%, and 125%, respectively. It can be evidently observed that these methods more or less involve complex secondary treatment on the surface of PI fibers to achieve the ultimate goal of enhancing the interfacial adhesion, which will inevitably lead to a series of issues that hinder the industrialization of corresponding technology, such as energy consumption, manufacturing cost, environmental protection, etc. Hence, it has become a challenging task to explore a simple, efficient, low-cost, and promising industrial surface-modification technology for PI fibers.

Compared to other aromatic fibers, such as Kevlar^®^, M5^®^, and Zylon^®^ [4,21,22], aromatic PI fibers possess copious designability of the molecular chain structure, i.e., the introduction of specific groups into the polymer main chain to improve the comprehensive properties of the fibers, such as 2-(4-aminophenyl)-6-amino-4(3H)-quinazolinone (AAQ), 2-(4-aminophenyl)-5-amino-benzimidazole (BIA), 5-amino-2-(2′-hydroxy-4′-aminophenyl)-benzoxazole (HBOA), 2,2′-bis(trifluoromethyl)-4,4′-diaminobiphenyl (TFMB), *p*-phenylenediamine (*p*-PDA), etc. In detail, Niu et al. [23] designed and synthesized an aromatic heterocyclic diamine AAQ containing the -NH- group, which was incorporated into the BPDA/*p*-PDA molecular backbone. The tensile strength and modulus of PI fibers increased significantly from the initial 1.2 GPa and 64.6 GPa to 2.8 GPa and 115.2 GPa, respectively. Bao et al. [24] introduced an HBOA diamine containing an -OH group into the BPDA/PRM macromolecular chain; meanwhile, the tensile strength and modulus of resultant fibers surprisingly reached 4.15 GPa and 125.93 GPa, respectively. To meet the requirements of wave-transparent composite reinforcement, Wang et al. [25] selected the TFMB diamine-containing -CF_3_ group and then introduced it into the BPDA/*p*-PDA/BIA polymer chain. The prepared PI fibers not only exhibited excellent mechanical properties (tensile strength of 2.62 GPa and modulus of 96.78 GPa), but also possessed a dielectric constant as low as 2.433 at 10 GHz. It is not difficult to see that by introducing specific groups into the molecular chain, the mechanical or dielectric properties of PI fibers have been significantly improved. However, the related investigation on advancing the surface activity of PI fibers based on this method has not been reported.

Epoxy resin with excellent mechanical properties and heat resistance is an ideal resin matrix for manufacturing FRPs with high mechanical strength. Additionally, the 3D cross-linking network structure of the epoxy molecular chain will produce a large number of active -OH groups during the curing process [26,27,28]. Based on these reasons, in order to improve the interfacial adhesion of PI fiber-reinforced epoxy composites, the 4,4′-diamino-(1,1′-biphenyl)-3,3′-diol (HAB) diamine monomer containing bis-OH group was selected as the fourth monomer to introduce the BPDA/*p*-PDA/BIA polyimide molecular chains in this work, which has been shown to significantly improve the cathode–electrolyte interface stability of lithium-ion batteries due to its rich chemical active sites for the polymer chains [29], and then, five kinds of PI fibers with different diamine ratios were manufactured through typical wet spinning technology. Effects of the HAB contents on the molecular chain interaction, aggregation structure, internal defects, and thermal and mechanical properties of the fibers were studied systematically. Moreover, the interfacial adhesion and its mechanism of unidirectional PI fiber/epoxy resin composites (PI/EPs) were also researched.

## 2. Materials and Methods

### 2.1. Materials

BPDA and BIA were supplied by Shijiazhuang Haili Chemical Co., Ltd. (Hebei, China) and purified before utilization. *p*-PDA was supplied by Changzhou Rongtuo Chemical Co., Ltd. (Jiangsu, China) and used directly. HAB was purchased from Bide Pharmatech Ltd. (Shanghai, China) and purified before use. N,N-dimethylacetamide (DMAc) was obtained from Tianjin Fuchen Chemical Reagent Factory (Tianjin, China) and utilized after distillation. Epoxy resins (E-51) and the curing agent (PA651) were supplied by Shanghai Macklin Biochemical Co., Ltd. (Shanghai, China) and utilized without further treatment.

### 2.2. Preparation of BPDA/p-PDA/HAB/BIA (BPHB) Polyimide Fibers

**Scheme 1:**
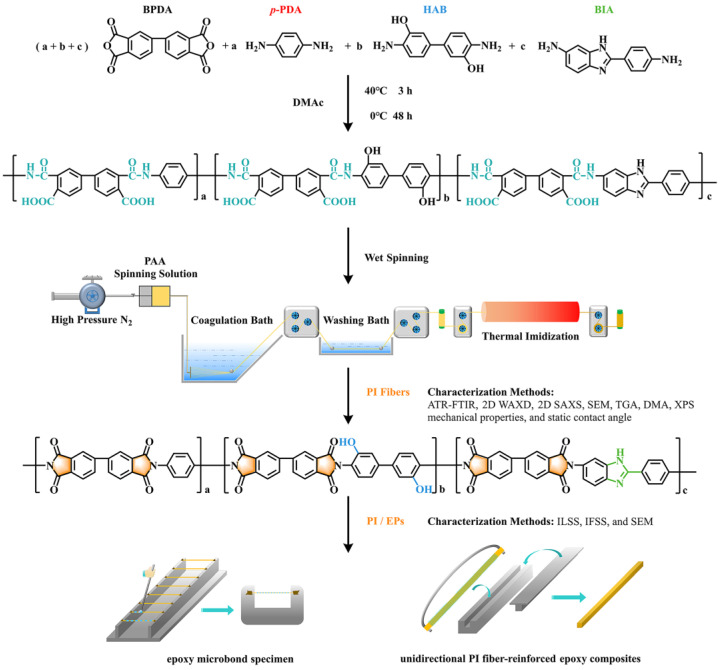
Fabrication routes of PI fibers and PI/EPs.

In the present work, the PI fibers were fabricated via a typical wet spinning technology, and their preparation route together with the chemical structure are displayed in Scheme 1. The BPDA/*p*-PDA/HAB/BIA poly(amic acid) (PAA) solutions were polymerized by dissolving diamines (*p*-PDA, HAB, and BIA) in DMAc solvent under a nitrogen atmosphere. After adding equimolar BPDA, the copolymerization was carried out at 40 °C for 4 h along with 0 °C for 48 h, and then, a brown spinning solution with a 16 wt % solid content was acquired. According to this procedure, all PAA resins with various *p*-PDA/HAB/BIA molar ratios (7:0:3, 6:1:3, 4:3:3, 2:5:3, and 0:7:3) were successfully synthesized.

After being filtrated and degassed under vacuum at 40 °C for 12 h, the above spinning solutions were extruded into the H_2_O/DMAc coagulation bath through a spinneret (100 holes, 75 μm in diameter) under nitrogen at 0.3 MPa; meanwhile, the as-spun fibers with a drawing ratio of 1.15 were produced. The PAA fibers were further washed with deionized water at 25 °C to remove residual DMAc from the filament. Afterward, these fibers were transferred into a series of high-temperature tube ovens at 100, 280, and 350 °C with a stretching ratio of 1.2 on the spinning rollers and then converted into the corresponding PI fibers. Finally, the annealed fibers were denoted as BPHB–0, 1, 3, 5, and 7, respectively, depending on the molar ratio of HAB in mixed diamines.

### 2.3. Preparation of PI Fiber-Reinforced Epoxy Composites

Unidirectional PI/EPs with the 55% volume fraction of reinforced fibers were fabricated using hand lay-up followed by compression molding [30]. The length of PI fibers used in the manufacturing of composites was first calculated, and then, these fixed-length fibers were arranged in parallel on a customized metal framework under pre-tension conditions. After being sufficiently impregnated in the uncured epoxy matrix with an E-51/PA651 mass ratio of 100/50, the impregnated fibers were delivered into a mold coated with the release agent and further cured at 60 °C for 1 h and 200 °C for 1 h under 0.2 MPa pressure. Subsequently, the unidirectional composites were taken out from the mold, cooled to room temperature, and cut into specimens with a width of 6 mm along with a thickness of 2 mm, suitable for the interlaminar shear strength test. In addition, the epoxy micro-bonded samples for the monofilament pull-out test were produced through a point-coating approach [18], in which the ingredient and curing procedure of epoxy were the same as those of PI/EPs. Scheme 1 concisely illustrates the preparation process of these two materials.

### 2.4. Characterization Methods

The intrinsic viscosities of PAA were evaluated using a SCHOTT 52510 Ubbelohde viscometer in a circulating water bath at 35 °C. Single-fiber mechanical properties were determined on a YM-06B instrument. The gauge length and extension rate were set to 20 mm and 10 mm min^−1^, respectively, and at least 20 single filaments were tested for each kind of fiber. Attenuated total reflectance Fourier transform infrared spectroscopy (ATR-FTIR) of PI fibers was performed in the range of 400–4000 cm^−1^ using a Nexus 670 instrument with a resolution of 4 cm^−1^. Thermo-gravimetric analyses (TGA) were carried out on a TA Q50 instrument in the range of 50–800 °C at a heating speed of 10 °C mim^−1^ under a nitrogen environment. Dynamic mechanical analyses (DMA) were conducted on a TA Q800 system with a frequency of 1 Hz and heating rate of 5 °C mim^−1^ from 50 to 400 °C.

Two-dimensional wide-angle X-ray diffraction (2D WAXD) was carried out on a Rigaku HomeLab instrument equipped with Hypix-6000 as a 2D detector and Cu kα (*λ* = 1.5405 Å) radiation, and then, FIT 2D software was utilized to process 2D WAXD data. The order degree (X) of the macromolecule can be obtained using the equation:

(1) $$X=\frac{U_{0}}{I_{0}}\times \frac{U_{X}}{I_{X}}\times 100\%$$

where U_0_ and U_X_ are the background of the reference sample and experimental sample, and I_0_ and I_X_ represent the integrated intensity of the diffraction line of the reference specimen and experimental specimen, respectively [31]. The orientation factor (f) along the fiber axial direction is estimated using Herman′s equation:

(2) $${f=(3\;<\cos}^{2}\psi >\;-\;1)/2$$

where *ψ* is the average angle between the fiber axis and the c axis of the crystal cell. In a given diffraction plane, <cos^2^*ψ>* represents an orientation parameter according to Wilchinsky′s theory [32], which can be calculated via the equation:

(3) $$\cos^{2}\psi =\frac{\int_{0}^{\pi /2}{I(\psi )\;\sin\psi \;\cos}^{2}\psi \;d\psi }{\int_{0}^{\pi /2}I(\psi )\;\sin\psi \;d\psi }$$

where I(*ψ*) is denoted as the intensity of a given diffraction plane (*hkl*).

Two-dimensional small-angle X-ray scattering (2D SAXS) was performed on a SAXS Xuess2-0 system equipped with Pilatus 200k as a 2D detector and Cu kα (*λ* = 1.5405 Å) radiation. The sample-to-detector distance and exposure time for each scattered sample were set to 1041 mm and 600 s, respectively. The internal microvoids radius (*R*) of PI fibers can be obtained using the Guinier function:

(4) $${I(q)=I}_{0}\;\exp(\frac{{-q}^{2}R^{2}}{5})$$

where *q* (*q* = 4π sin*θ*/*λ,* and *λ* is the wavelength) represents the scattering vector, *R* means the radius of internal defects on the circle cross-section, and I_0_ and I(*q*) are the reciprocal of the incident intensity and scattering intensity, respectively [33]. The Fankuchen successive tangent method is utilized to deduce the multi-level sizes of microvoids, and then, the mean radius (*R*) of microvoids is effectively calculated according to the equation:

(5) $$R=\sum R_{i}\omega_{i}\;(i=1,\;2,\;3\text{…})$$

where R_i_ and *ω*_i_ are the radius and volume fraction of microvoids with multi-level sizes in the successive tangent method [34].

X-ray photoelectron spectroscopy (XPS) was conducted on a Thermo Kalpha instrument with monochromatic Al kα radiation. The water static contact angle was determined with a JC2000D2M contact angle goniometer. An Olympus BX51 optical microscope equipped with a Sony CCD IRIS digital camera was utilized to monitor the optical microscopic images of the epoxy micro-bonded sample, and after that, the epoxy static contact angles of PI fibers were measured by importing the corresponding optical microscopic photos into ImageJ software.

According to GB3357-82, the interlaminar shear strength (ILSS) values of unidirectional PI/EPs were conducted based on a three-point short-beam bending test on a SANS CMT 4104 universal material testing instrument, and then, the corresponding ILSS value was calculated through the equation:

(6) $$\mathrm{ILSS}=\frac{{3P}_{b}}{4bh}$$

where P_b_ is the maximum compressive load force, b and h are the width and the thickness of specimen, respectively.

The interfacial shear strength (IFSS) values of epoxy micro-bonded specimens were assessed on an HM410 composites interfacial evaluation instrument. Epoxy microspheres with a diameter of 50 to 90 μm were selected as test objects, and the IFSS values were calculated according to the formula:

(7) $$\mathrm{IFSS}=\frac{F_{\max}}{\pi dL}$$

where F_max_ represents the maximum load force, *d* and L are the diameter of the fiber and the length of microsphere, respectively.

The morphology for the interlaminar shear failure, epoxy micro-bonded destruction, and fiber surface and internal structures were recorded on a JSM-7800F scanning electron microscope (SEM) instrument. All specimens were coated with Pt about 5 nm before observation.

## 3. Results and Discussion

### 3.1. Mechanical Properties

Table 1 summarizes the detailed values for the intrinsic viscosity of PAA solutions, as well as the mechanical properties of corresponding PI fibers, and the typical tensile strength–elongation curves of resultant fibers are plotted in Supplementary Figure S1. It can be observed that the BPHB–0 fiber without HAB moieties possesses tensile strength of 2.32 GPa and modulus of 118.51 GPa. Taking the BPHB–0 fiber as a reference, the tensile strength of BPHB fibers first increases and then decreases after the addition of HAB, while their modulus gradually decreases from 106.60 GPa of BPHB–1 fiber to 72.61 GPa of BPHB–7 fiber. Particularly, the BPHB–3 fiber possesses the outstanding strength and modulus of 2.62 GPa and 100.15 GPa, respectively, which proves that the high-strength and high-modulus PI fibers with hydroxyl groups had been successfully manufactured in the current work depending on the evaluation standard of the organic fiber [24].

Generally, the mechanical properties of synthetic fibers depend on their structural parameters, including intrinsic viscosity, intra-/intermolecular interactions, and structure defects, along with the molecular chain orientation and packing (aggregation structure) [35,36,37]. As listed in Table 1, increasing the HAB content results in a decrease in the inherent viscosity of the spinning solution from 3.14 to 2.04 dL g^−1^, meaning that the intrinsic viscosity is not the leading reason for the improvement in mechanical properties of the final BPHB fibers. In addition, these PI fibers possess different chemical structures owing to the incorporation of various *p*-PDA/HAB/BIA molar ratios, and thus, the evolution of microstructures has become the investigation focus, analyzing the changes in mechanical properties, i.e., the influence of intra-/intermolecular associations, aggregation structure, and inner defects.

### 3.2. Chemical Structure and Hydrogen-Bonding Interaction

**Figure 1:**
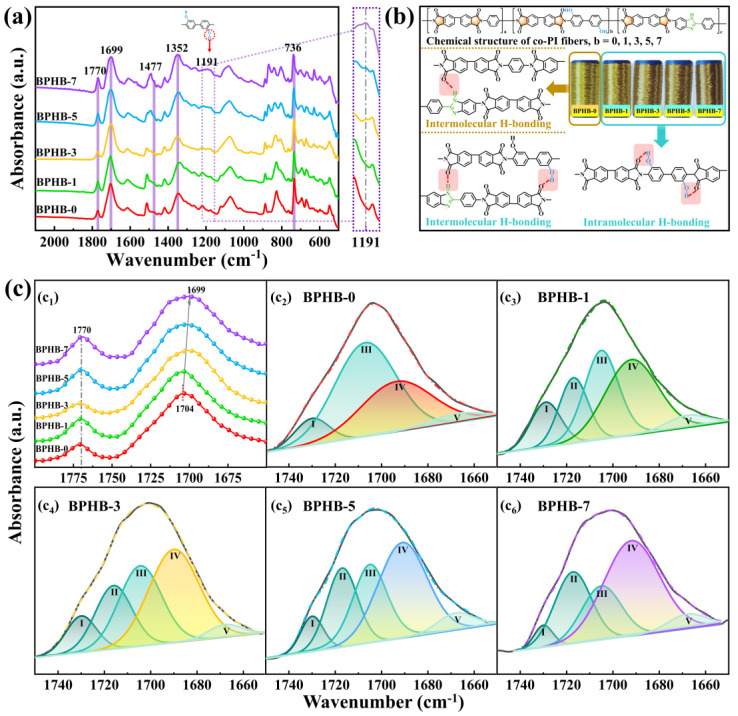
(**a**) ATR–FTIR spectra of the PI fibers with the different *p*-PDA/HAB/BIA molar ratios. (**b**) Intra-/intermolecular H-bonding interactions in BPHB fibers. (**c_1_**) Magnified ATR–FTIR spectra of PI fibers at the wavenumber range of 1800–1650 cm^−1^; (**c_2_**–**c_6_**) curve-fitting ATR–FTIR spectra in the wavenumber range from 1650 cm^−1^ to 1750 cm^−1^.

The chemical structures of PI fibers with various *p*-PDA/HAB/BIA molar ratios were confirmed by ATR-FTIR. As shown in Figure 1a, four characteristic absorption bands of all the fibers are located at 1770 cm^−1^ (asymmetric stretching of C=O), 1704 cm^−1^ (symmetric stretching of C=O, namely, *V*_(sym)C=O_), 1352 cm^−1^ (axial stretching of C-N-C), and 736 cm^−1^ (bending of cyclic imide), respectively, indicating that the PI fibers with a high imidization degree had been successfully obtained. The absorption bands at 1477 cm^−1^ and 1191 cm^−1^ are ascribed to the C=N stretching of benzimidazole in BIA and the C-O stretching of Ar-OH in HAB [24,38]. Meanwhile, the intensity of the absorption peak at 1191 cm^−1^ simultaneously increases with increasing HAB content, implying the successful introduction of HAB moieties into the polymer chains.

Because of the existence of the -OH group in the HAB monomer and the -NH group in the BIA monomer, as shown in Figure 1b, intra-/intermolecular H-bonding interactions with the oxygen atom of the C=O groups on the imide ring as proton acceptors may be established in BPHB fibers. When more HAB is introduced, the peak position of *V*_(sym)C=O_ on cyclic imide continuously shifts from 1704 cm^−1^ to 1699 cm^−1^, as shown in Figure 1c_1_, i.e., the red shift, which is similar to that in the studies by Lian et al. showing that the red shift of C=O would occur upon the formation of H-bonding [39]. To evaluate the contribution degree of -OH to intra-/intermolecular H-bonding associations, curve-fitting ATR-FTIR spectroscopy in the range from 1650 cm^−1^ to 1750 cm^−1^ can be applied to quantitatively analyze H-bonding associations based on the previous studies [23,25]. As depicted in Figure 1c_2_–c_6_, four peaks are separately identified for the BPHB–0 fiber, and the BPHB–1, 3, 5, and 7 fibers have five peaks. Band I (about 1730 cm^−1^) and band V (1668 cm^−1^) are attributed to the symmetric and asymmetric stretching of C=O in the intermolecular chemical links [40]. Band II (around 1717 cm^−1^) and band IV (ca. 1690–1694 cm^−1^) correspond to the C=O symmetric stretching on the imide ring of intramolecular and intermolecular H-bonding associations, respectively [41]. Band III (near 1705 cm^−1^) is assigned to the C=O symmetric stretching on cyclic imide without H-bonding interactions [42]. For quantitative analysis, the proportion of intra-/intermolecular H-bonding imides, i.e., H_(intra)_ and H_(inter)_, can be calculated based on Equations (8) and (9) [43], respectively.

(8) $$H_{\left(\mathrm{intra}\right)}=\frac{S_{\mathrm{II}}}{S_{\mathrm{II}}{+S}_{\mathrm{III}}{+S}_{\mathrm{IV}}}\times \;100\%$$

(9) $$H_{\left(\mathrm{inter}\right)\;}=\frac{S_{\mathrm{IV}}}{S_{\mathrm{II}}{+S}_{\mathrm{III}}{+S}_{\mathrm{IV}}}\times \;100\%$$

where S_II_, S_III_, and S_IV_ denote the peak areas of bands II, III, and IV, respectively. Curve-fitted results of all deconvoluted bands are summarized in Table 2.

For the BPHB–0 fiber, H_(inter)_ was calculated to be 37.8% attributing to the interaction between the N-H group in BIA and the C=O group in the imide ring (Figure 1b), while the frequency shift of band IV is merely 13 cm^−1^. After the incorporation of HAB, intramolecular and intermolecular H-bonding interactions are formed in the corresponding BPHB fibers simultaneously. H_(intra)_ merely increases from 20.61% of BPHB–1 fiber to 25.98% of BPHB–7 fiber, which is suggested to be due to the combined effects of the non-coplanar conformation and the atomic distance, as illustrated in Supplementary Figure S2 [44,45]. In contrast, H_(inter)_ is significantly enhanced from 43.75% to 52.66%, and the wavenumber shift of band IV further increases to the range of 13–15 cm^−1^ (Table 2), indicating the formation of strong intermolecular H-bonding. Furthermore, it can be clearly observed that these calculated results not only commendably demonstrate the wavenumber red shift of *V*_(sym)C=O_ on cyclic imide, but also explain the significant improvement in the tensile strength of PI fibers after the introduction of HAB. However, when the content of HAB exceeds 50 mol %, the prepared fibers display reduced tensile strength, even if H_(inter)_ continuously increases. This phenomenon may be caused by the fact that a weak intermolecular H-bonding interaction formed in BPHB–5 and BPHB–7 fibers, as revealed through the relatively low-frequency shift of band IV. Consequently, the tensile strength of BPHB fibers is related not only to the percentage of intermolecular H-bonding, but also to the strength of intermolecular hydrogen bonding.

### 3.3. Aggregation Structure

**Figure 2:**
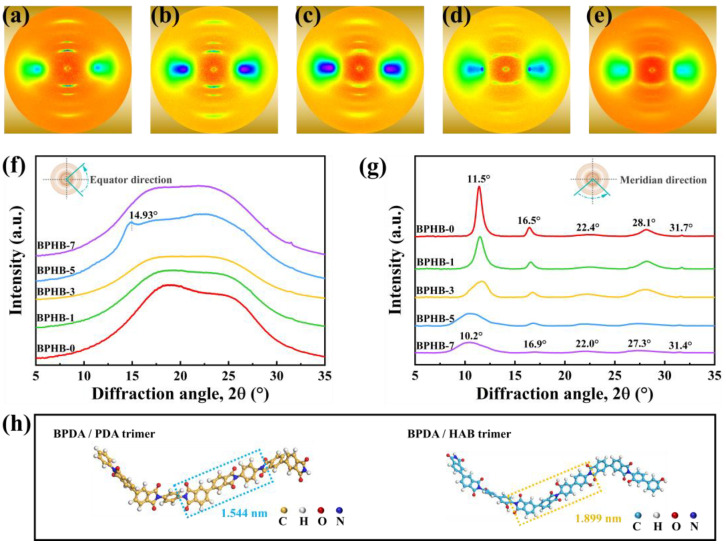
(**a**–**e**) 2D WAXD patterns for BPHB fibers with *p*-PDA/HAB/BIA molar ratios of 7/0/3, 6/1/3, 4/3/3, 2/5/3, and 0/7/3, respectively. (**f**) Equator and (**g**) meridian directions of the 1D WAXD profiles for the PI fibers with different *p*-PDA/HAB/BIA molar ratios. (**h**) Simulated conformations of BPDA/*p*-PDA and BPDA/HAB trimers, as well as the length of its repeat units, calculated by using the COMPASS force field of Materials Studio 8.0.

In addition to the strong H-bonding associations, the aggregation structure is usually considered to be another key factor governing the mechanical properties of PI fibers [46]. The variations of the ordered packing and orientation degree for the BPHB fibers were investigated by using the 2D WAXD technique, and the detailed information is exhibited in Figure 2a–e. Along the meridional direction, these PI fibers display distinct diffraction streaks, manifesting as highly oriented polymer chains parallel to the fiber axis being formed. However, the diffraction streaks are relatively diffuse in the equatorial direction, i.e., the amorphous halos, revealing the poor lateral packing of molecular chains along the transversal direction. Notably, the BPHB–5 fiber exhibits clear equatorial streaks (2*θ* = 14.93°) and several evident diffraction arcs in the quadrants (Figure 2d), confirming that it possesses a well-defined 3D crystalline structure, whereas the BPHB–7 fiber only shows a weak crystalline signal (Figure 2e). This phenomenon may be caused by the following two factors: firstly, the low reactive activity of HAB and the content of up to 50 mol % in mixed diamines are conductive to the formation of a series of long homopolymer blocks of BPDA/HAB units in the polyimide chain. Secondly, the aggregation structure of PI fibers affects the probability of the crystal structure with BPDA/HAB sequences.

To evaluate the microstructural information of molecular chains, the 1D WAXD profiles along the equatorial and meridional directions were obtained from the corresponding 2D WAXD patterns. Figure 2f displays the 1D WAXD intensity profiles along the equator; these BPHB fibers exhibit broad diffraction peaks in the range of 10–30°. Furthermore, their order degree (X) values of macromolecules were measured via Equation (1), which were calculated as 8.53 *U_0_/I_0_*, 6.72 *U_0_/I_0_*, 4.48 *U_0_/I_0_*, 5.67 *U_0_/I_0_*, and 6.89 *U_0_/I_0_*, respectively, corresponding to BPHB–0, 1, 3, 5, and 7 fibers. Apparently, the X values first drop until the HAB content increases to 30 mol %, and then rise with the further addition of HAB, which confirms the evolution of polymer chain regularity [10].

For the BPHB–0 fiber, five Bragg diffraction peaks at 2*θ* = 11.5°, 16.5°, 22.4°, 28.1°, and 31.7° can be identified in Figure 2g, revealing the highly oriented structure along the meridian direction. Moreover, the calculated *d*-spacing value for 2*θ* = 11.5° is 0.769 nm, which is close to one-half of the repeat unit length on the BPDA/*p*-PDA trimer (1.544 nm) at the minimum energy conformation (Figure 2h). Therefore, the diffraction peaks at 11.5° are ascribed to the (002) plane. Accordingly, the diffraction streaks at 2*θ* = 16.5°, 22.4°, 28.1°, and 31.7° can be attributed to the (003), (004), (005), and (006) planes. Similarly, the peaks at 2*θ* = 10.2°, 16.9°, 22.0°, 27.3°, and 31.4° for the BPHB–7 fiber are assigned to the (002), (003), (004), (005), and (006) planes, respectively, because the repeat length of the BPDA/HAB trimer (Figure 2h) is 1.899 nm. Obviously, the diffraction angle of the (002) plane gradually decreases from 11.5° (BPHB–0) to 10.2° (BPHB–7) with the increase in *d*-spacing (Table 3), implying that some BPDA/*p*-PDA units are replaced by larger BPDA/HAB units [25].

As illustrated in Figure 2g, the half-peak widths of the (002), (003), (004), (005), and (006) diffraction peaks become rather broad and diffuse with the incorporation of more HAB, demonstrating the evolution of the main chain orientation. The diffraction peak intensity of the (002) plane was applied to calculate the molecular orientation degree using Equations (2) and (3) [32]. In Table 3, the orientation factors of PI fibers drastically decrease from 90.79% (BPHB–0) to 46.48% (BPHB–7). This may be because the increasing H-bonding associations, when more HAB is incorporated into the polymer chains of PAA, hinder the rearrangement of the PAA molecular chain along the fiber axis during the wet-spinning process, which leads to the low orientation factors of corresponding PI fibers prepared under the hot-stretching ratio of 1.2. Evidently, the decreasing tendency of the orientation factor is similar to that of the modulus for BPHB fibers, revealing that the decrease in the modulus mainly originated from the reduction in molecular orientation along the fiber axis. In addition, the BPHB–0, 1, and 3 fibers possess an orientation degree greater than 70%, which guarantees the good mechanical properties of the final BPHB fibers.

### 3.4. Morphology and Microvoids Analyses

**Figure 3:**
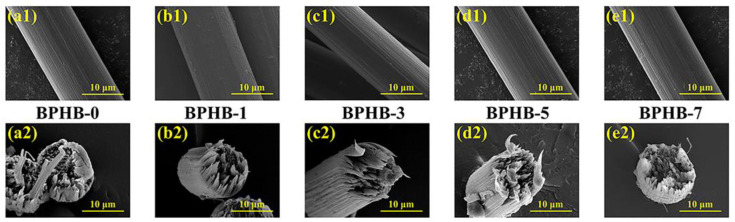
SEM images of the surface (**a_1_**–**e_1_**) and cross-section (**a_2_**–**e_2_**) of PI fibers.

The various diamine ratios also affect the morphologies and internal defects of PI fibers and then regulate the mechanical performances of the fibers [10]. Here, the surface and fracture morphologies of BPHB fibers could be visually observed by SEM. As presented in Figure 3a_1_–e_1_, all fibers possess dense surface morphologies and relatively uniform diameters of about 12.5 μm, while some continuous gullies can be identified along the fiber axis, which is caused by the spinneret during the spinning of PAA fibers [8]. Figure 3a_2_–e_2_ show the cross-sectional images of resultant fibers; these PI fibers show lamellar fibrillate structures, and further observation illustrates that the lamellar fibrils on the skin of fibers are more than those on the core section, i.e., the “skin-core” structure [46]. Due to the preparation of samples mediated by a transverse fracture in liquid nitrogen, the laminar fibrils of the skin exhibit serious deformation, indicating the high fibril degree along the fiber drawing direction and the excellent toughness of BPHB fibers. It is noteworthy that the whole cross-sectional morphology of the BPHB–3 fiber displays the features of a homogenous laminar fibril distribution and disappearance of the boundary between the outer skin and inner core (Figure 3c_2_), which is the embodiment of excellent mechanical properties and dense internal structures of PI fibers. However, the BPHB–7 fiber exhibits rather flat cross-sections (Figure 3e_2_), indicating a brittle fracture behavior accounting for its poor mechanical properties.

**Figure 4:**
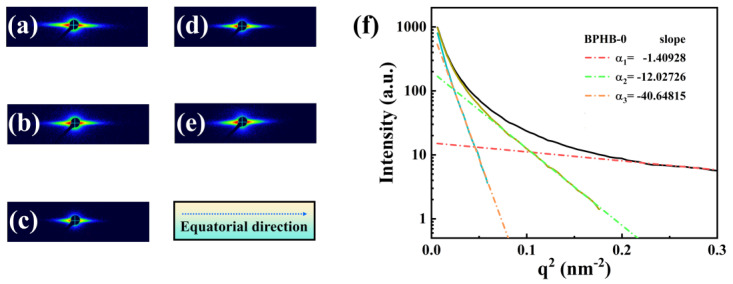
(**a**–**e**) 2D SAXS patterns for BPHB fibers with *p*-PDA/HAB/BIA molar ratios of 7/0/3, 6/1/3, 4/3/3, 2/5/3, and 0/7/3, respectively. (**f**) Guinier plot of the BPHB–0 fiber along the equator direction via Guinier functions and the Fankuchen successive tangent method.

Depending on the analysis results of the cross-sectional morphologies of BPHB fibers, it is necessary to investigate the evolution of internal defects in the fibers after the introduction of HAB. Normally, due to the intense substance exchange during the coagulation and thermal-imidization processes in the wet-spinning method [38], some porous structures are inevitably produced in the final PI fibers and further influence their mechanical properties. These microvoids can be evaluated by using the 2D SAXS technique [37]. As shown in Figure 4a–e, the 2D SAXS patterns of BPHB fibers present a sharp and needle-shaped scattering streak along the equatorial direction, indicating that these microvoids are orderly aligned parallel to the fiber axis.

Figure 4f displays the Guinier plot of the BPHB–0 fiber in the equator by using Guinier functions (Equations (4) and (5)) and Fankuchen’s method [33,34], illustrating that the radius of microvoids in BPHB fibers exhibits multi-level distributions. Meanwhile, the detailed radius values of corresponding PI fibers are also listed in Table 3. The average radius decreases at first with the increase in the HAB content, from 0 mol % to 30 mol %, and then increases gradually as the content of HAB continues to increase. Among all fibers, the BPHB–3 fiber exhibits a relatively narrow radius distribution of 1.773, 6.729, and 11.987 nm, and its average microvoids radius is the smallest (8.486 nm), which is consistent with the results based on the fracture morphology and demonstrates a denser inner structure formed in the fiber. Consequently, the BPHB–3 fiber shows excellent mechanical properties. Moreover, the radius distribution (2.398, 7.323, and 13.626 nm) and the average radii (9.730 nm) of microvoids for the BPHB–7 fiber are relatively large compared to those of the BPHB–3 fiber, which is a critical element impeding the enhancement of its mechanical properties. Ultimately, it can be concluded that the reduction in the microvoid radius cannot be neglected to advance the mechanical properties of BPHB fibers.

### 3.5. Thermal Properties

**Figure 5:**
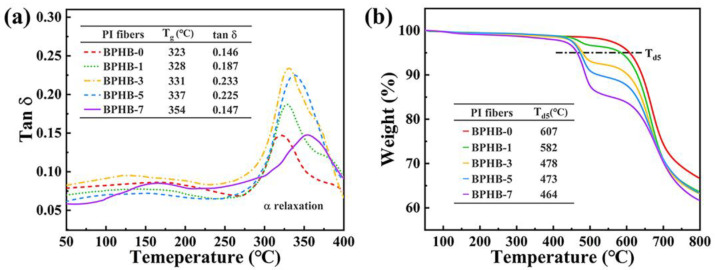
(**a**) DMA curves and (**b**) TGA curves of the PI fibers with different *p-*PDA/HAB/BIA molar ratios.

Figure 5a illustrates the DMA curves with the temperature ranging from 50 to 400 °C. The glass transition temperatures (T_g_) of BPHB fibers are gradually enhanced from 323 °C for the BPHB–0 fiber to 354 °C for the BPHB–7 fiber, which follows the increased H-bonding imides after the addition of more HAB. In addition, it should be noted that the segmental motion must correspond to the intensity of the α relaxation peak (i.e., the value of tan *δ*), and the lateral packing order of macromolecules in BPHB fibers also significantly affect the segmental motion ability [23], as indicated based on the 1D WAXD in Table 3. Consequently, the tan *δ* values of PI fibers show an increasing–decreasing tendency.

The thermal stabilities of BPHB fibers were measured via TGA under a nitrogen environment. In Figure 5b, the 5%-weight-loss temperatures (T_d5_) of resulting fibers are in the range of 464–607 °C. Obviously, when HAB moieties are introduced into the BPHB/*p*-PDA/BIA molecular chains, the TGA curves have undergone substantial changes from the one-step degradation of BPHB–0 fiber to the two-step degradation of the fiber containing HAB groups, revealing that the incorporation of the HAB moiety presents a negative effect on the thermal stability of PI fibers.

### 3.6. Surface Chemical Activity in BPHB Fibers

**Figure 6:**
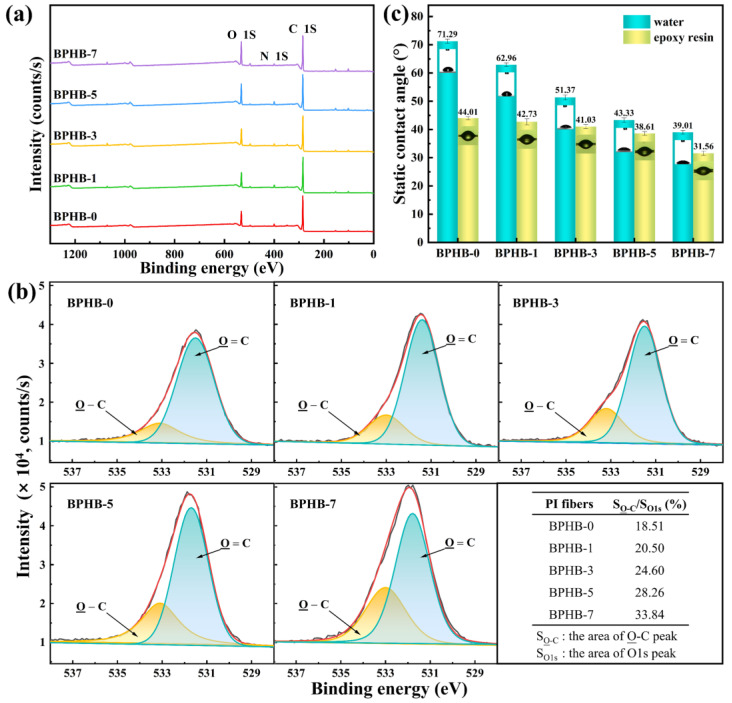
(**a**) XPS wide-scan spectra, and (**b**) curve-fitting O1s high-resolution XPS spectra for the BPHB fibers. (**c**) The static contact angle of water and epoxy resin for the prepared fibers.

The XPS and static contact angle characterizations were utilized to quantitatively analyze the surface activity of BPHB fibers. Figure 6a shows the XPS comparison of these fibers, and the binding energy at 285.08, 400.00, and 532.08 eV is distributed to C_1s_, N_1s_, and O_1s_, respectively. Further, the surface element contents of the corresponding fibers were also accurately measured, as listed in Supplementary Table S1, and the O/C value significantly advances from 0.19 for the BPHB–0 fiber to 0.30 for the BPHB–7 fiber. Furthermore, curve-fitting O_1s_ high-resolution spectra were determined using XPSPEAK software. In Figure 6b, the binding energy at 533.08 and 531.78 eV are identified as O-C and O=C, respectively [19], and then, the ratio of the area for the O-C peak to the area for the O_1s_ peak is defined as S_O__-C_/S_O1s_. For the BPHB–0 fiber, the S_O__-C_/S_O1s_ value was calculated to be 18.51%, which originated from the intermolecular chemical links, as revealed in Figure 1c. After the introduction of HAB, the S_O__-C_/S_O1s_ values of BPHB–1, 3, 5, and 7 fibers are 20.50%, 24.60%, 28.26%, and 33.84%, respectively, which is mainly due to the increase in the Ar-OH content compared to that in the BPHB–0 fiber. Hence, this phenomenon confirms that BPHB fibers with different surface activities were successfully produced.

When the content of HAB in molecular chains increased from 10 mol % to 70 mol %, as shown in Figure 6c, the static contact angles of water and epoxy resin for PI fibers are dramatically reduced from 62.96° and 42.73° for the BPHB–1 fiber to 39.01° and 31.56° for the BPHB–7 fiber, while those of the BPHB–0 fiber without HAB is 71.29° and 44.01°, respectively. These test results clarify that the introduction of HAB can not only enhance the surface activity of the prepared fiber, but also significantly improve the interfacial wettability between the PI fiber and epoxy.

### 3.7. Interfacial Properties of BPHB Fiber-Reinforced Epoxy Composites

**Figure 7:**
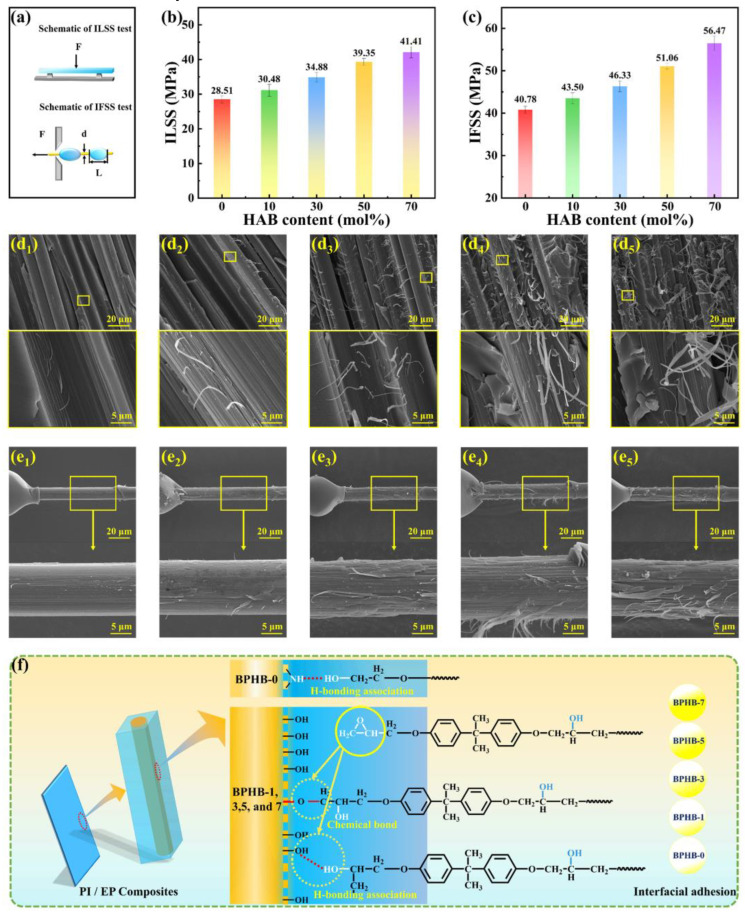
(**a**) Schematic of ILSS and IFSS test. (**b**) ILSS values and (**c**) IFSS values for the BPHB fibers. (**d_1_**–**d_5_**) Fracture images of the ILSS test, and (**e_1_**–**e_5_**) failure images of the IFSS test for the PI/EPs, in which the fibers have *p*-PDA/HAB/BIA molar ratios of 7/0/3, 6/1/3, 4/3/3, 2/5/3, and 0/7/3, respectively. (**f**) Interfacial interaction mechanism of the unidirectional PI/EPs.

The influence of HAB contents on the interface properties of unidirectional PI/EPs were quantitatively evaluated by using ILSS and IFSS tests, as in Figure 7a. In detail, the ILSS and IFSS for the PI fiber without the HAB moiety are merely 28.51 and 40.78 MPa, as shown in Figure 7b–c, and then increase to 41.41 and 56.47 MPa with the HAB content of 70 mol %, respectively. Compared with the content of 0 mol %, the ILSS and IFSS for the BPHB–7 fiber increase by 45.25% and 38.47%, in which the IFSS value is already higher than that of plasma-modified PI fibers listed in Table 4. Moreover, the values for the BPHB–3 fiber increased by 22.34% and 13.61% as well.

Furthermore, SEM technology was applied to investigate the interface failure modes of ILSS test samples (Figure 7d_1_–d_5_). As shown in Figure 7d_1_, the overall appearance of the fiber in unidirectional composites is relatively intact, which indicates that the failure form comprises the destruction of resin matrix originating from the poor interfacial load transfer effect caused by the weak bonding strength between the fiber and resin. When the HAB content in fiber is 10 mol % and 30 mol %, the surface of reinforced fibers exhibits an increasing fibril phenomenon, as in Figure 7d_2_–d_3_, reflecting that interfacial debonding present in the failure mode, i.e., the fibers gradually play a role of bearing the load during the process of material failure. After the content reaches 50 mol %, interfacial debonding has become the main failure mode, i.e., the reinforcement displays its reinforcing and toughening effects, which can be seen from the denser fibril structure and the adhesive bulk resin matrix on the fiber surface in Figure 7d_4_–d_5_. Consequently, with the increase in HAB in BPHB fibers, the interface failure mode of unidirectional PI/EPs gradually changes from the matrix failure mode to the reinforcement failure mode. This interface enhancement rule is more intuitively verified in the interface failure morphologies of the IFSS test samples (Figure 7e_1_–e_5_).

The difference in interfacial interaction is the predominant factor affecting the interfacial properties of unidirectional PI/EPs. Comparing Figure 3a_1_ and Figure 7e_1_, owing to the poor interfacial bonding force originating from the weak H-bonding interaction, as shown in Figure 7f (the bond energy of -NH⋯O is generally lower than that of -OH⋯O [24]), and van der Waals forces [18], the failure morphology of the epoxy micro-bonded specimen for the BPHB–0 fiber exhibits slight cortical tearing behaviors. For BPHB–1,3,5, and 7 fibers, the incorporation of HAB into the polyimide molecular chain has brought about significant changes (Figure 7f) in two aspects: on one hand, the -OH groups in the fiber surface, as active sites, possess the probability of a ring-opening reaction with the epoxy end groups to form a stable interface chemical bond [47]. On the other hand, these reactive -OH groups in the interfacial phase tend to generate strong H-bonding associations [17]. As revealed through the tearing of the cortex in Figure 7e_2_–e_5_, the increase in HAB molar ratio contributes to advancing the interfacial adhesion between the fiber and epoxy matrix, which is helpful to substantially improve the strengthening and toughening effect of the reinforced fiber in the composites.

## 4. Conclusions

In this work, through the introduction of an HAB moiety, PI fibers with high tensile strength and excellent interfacial adhesion were successfully fabricated via wet-spinning technology. Curve-fitting ATR–FTIR spectroscopy confirmed the variation in H-bonding associations, and the fiber with a *p*-PDA/HAB/BIA molar ratio of 4/3/3 displayed the highest wavenumber shift of 15 cm^−1^. Meanwhile, 2D SAXS and SEM results indicated that the narrow radius distribution of microvoids in fibers contributed to forming a dense internal structure. 2D WAXD showed that the introduction of HAB destroyed the orientation degree of macromolecules along the fiber axis. These results clarified that the BPHB fibers with strong intermolecular H-bonding, lower radii of microvoids, and higher molecular chain orientation will result in better mechanical properties. Therefore, the BPHB–3 fiber possesses excellent mechanical properties, with strength and modulus of 2.62 and 100.15 GPa, respectively.

The value of S_O__-C_/S_O1S_ increased from 18.51% for the BPHB–0 fiber to an impressive 33.84% for the BPHB–7 fiber, indicating that the number of active -OH groups on the fiber surface was also increased synchronously with the increase in HAB content of polyimide chains. For PI/EPs, the chemical active sites (-OH groups) of fiber surfaces could generate stable chemical bonds and strong intermolecular H-bonding associations with the epoxy matrix at the interface phase. Under the combined effects of these two interfacial interactions, the ILSS and IFSS values of unidirectional PI/EPs with BPHB–3 fiber reached 34.88 and 46.33 MPa, respectively, compared to the values of the BPHB–0 fiber.

Consequently, by introducing HAB moiety into the polyimide molecular chain, high-performance PI fibers with high tensile strength and excellent interfacial properties were successfully manufactured in this study, which has broad application potential in the field of PFRPs.

## Figures and Tables

**Table 1:** Intrinsic viscosities of the PAA solutions and the mechanical properties of corresponding PI fibers.

PI Fibers	*p*-PDA:HAB:BIA (Molar Ratio)	Intrinsic Viscosity (dL g^−1^)	Tensile Strength (GPa)	Modulus (GPa)	Elongation (%)
BPHB–0	7:0:3	3.14	2.32 ± 0.16	118.51 ± 7.83	1.95 ± 0.18
BPHB–1	6:1:3	2.86	2.37 ± 0.11	106.60 ± 10.1	2.60 ± 0.20
BPHB–3	4:3:3	2.45	2.62 ± 0.10	100.15 ± 7.33	3.30 ± 0.22
BPHB–5	2:5:3	2.13	2.26 ± 0.13	87.60 ± 8.61	3.30 ± 0.28
BPHB–7	0:7:3	2.04	1.64 ± 0.11	72.61 ± 9.70	2.65 ± 0.25

**Table 2:** Calculated results of curve-fitting ATR–FTIR spectra in the wavenumber range from 1650 cm^−1^ to 1750 cm^−1^.

PI Fibers	Intermolecular Chemical Links				Cyclic Imide							H_(intra)_	H_(inter)_
	Band I		Band V		Band II		Band III		Band IV				
	Freq. (cm^−1^)	Area (%)	Freq. (cm^−1^)	Area (%)	Freq. (cm^−1^)	Area (%)	Freq. (cm^−1^)	Area (%)	Freq. (cm^−1^)	Area (%)	Shift (cm^−1^)	(%)	(%)
BPHB–0	1730	8.34	1668	2.03	-	-	1707	55.75	1694	33.88	13	-	37.80
BPHB–1	1729	12.38	1668	4.77	1717	17.08	1705	29.52	1691	36.25	14	20.61	43.75
BPHB–3	1730	9.15	1668	3.02	1716	19.72	1705	29.00	1690	39.11	15	22.45	44.53
BPHB–5	1730	7.15	1668	4.37	1717	21.88	1705	24.90	1691	41.70	14	24.73	47.13
BPHB–7	1730	3.77	1668	2.88	1717	24.25	1705	19.94	1692	49.16	13	25.98	52.66

**Table 3:** 1D WAXD and 1D SAXS results of PI fibers with various molar ratios of diamines.

PI Fibers	1D WAXD				1D SAXS						
	Order Degree	2θ (°)	d-spacing	Orientation Factors (%)	R_1_ (nm)	R_2_ (nm)	R_3_ (nm)	ω_1_ (%)	ω_2_ (%)	ω_3_ (%)	$\bar{R}$ (nm)
BPHB–0	8.53 *U_0_/I_0_*	11.50	0.769	90.79	2.654	7.754	14.256	19.03	28.08	52.89	10.222
BPHB–1	6.72 *U_0_/I_0_*	11.53	0.767	85.53	2.369	7.284	13.940	17.06	28.45	54.49	10.072
BPHB–3	4.48 *U_0_/I_0_*	11.24	0.787	74.04	1.773	6.729	11.987	21.00	25.79	53.21	8.486
BPHB–5	5.67 *U_0_/I_0_*	10.57	0.836	62.14	2.279	7.246	13.190	23.23	26.07	50.07	9.023
BPHB–7	6.89 *U_0_/I_0_*	10.23	0.864	46.48	2.398	7.323	13.626	18.81	28.31	52.88	9.730

**Table 4:** IFSS values of the different modification methods for FRPs.

Composites		Modification Technology		IFSS (MPa)	Ref.
Fibers	Matrix	Method	Element		
Aramid	BMI ^a^	plasma	O_2_	49.35	[48]
CF	EP ^b^	surface grafting	CNTs ^c^	88.18	[49]
UHMWPE	EP ^b^	plasma	He	18.78	[50]
PBO	EP ^b^	plasma	O_2_	55.80	[51]
PI	EP ^b^	functional groups of backbone	HAB, 30 mol %	46.33	This work
			HAB, 70 mol %	56.47	This work
PI	EP ^b^	reinforcing particle	HBPSi ^d^	33.20	[52]
PI	EP ^b^	waterborne sizing	epoxy	31.87	[18]
PI–1	EP ^b^	coupling agent	silane	46.20	[20]
PI–2	EP ^b^	coupling agent	silane	43.00	[20]
PI	PAA ^e^	S–P ^f^	Silane, O_2_	38.82	[16]
PI	PU ^g^–EP ^b^	plasma	O_2_	56.03	[17]

^a^ bismaleimide;^b^ epoxy; ^c^ carbon nanotubes; ^d^ hyperbranched poly-siloxane; ^e^ poly(amic acid); ^f^ silane coupling agent-plasma; ^g^ polyurethane.

## Data Availability

The data presented in this study are available upon request from the corresponding author, based on reasonable requirements.

## Supplementary Materials

The following supporting information can be downloaded at: https://www.mdpi.com/article/10.3390/polym15041032/s1, Figure S1: Typical tensile strength–elongation curves of the PI fibers with various diamine molar ratios; Figure S2: Simulated results of the dihedral angle between the imide ring and benzene ring containing -OH groups, as well as the atomic distance between the H atom of -OH and O atom of the adjacent imide ring carbonyl for the BPHB/HAB polymer chain in the lowest energy conformation; Table S1: Surface elemental compositions of BPHB fibers with various diamine molar ratios.
