# Supplementary material for: Preparation and Interfacial Properties of Hydroxyl-Containing Polyimide Fibers
Source: Polymers (Basel). 2023 Feb 19;15(4):1032. doi: 10.3390/polym15041032 (PMC9967378; doi:10.3390/polym15041032)
Supplement: Supplementary file 1 [file polymers-15-01032-s001.zip › polymers-2144863-supplementary.pdf]

polymers

Article

Preparation and Interfacial Properties of Hydroxyl-Containing Polyimide Fibers

Jiang Du, Chuanzhi Pu, Xianyu Sun, Qi Wang, Hongqing Niu * and Dezhen Wu

State Key Laboratory of Chemical Resource Engineering, School of Materials Science and Engineering, Beijing University of Chemical Technology, Beijing 100029, China
* Correspondence: niuhq@mail.buct.edu.cn

Supplementary Files:

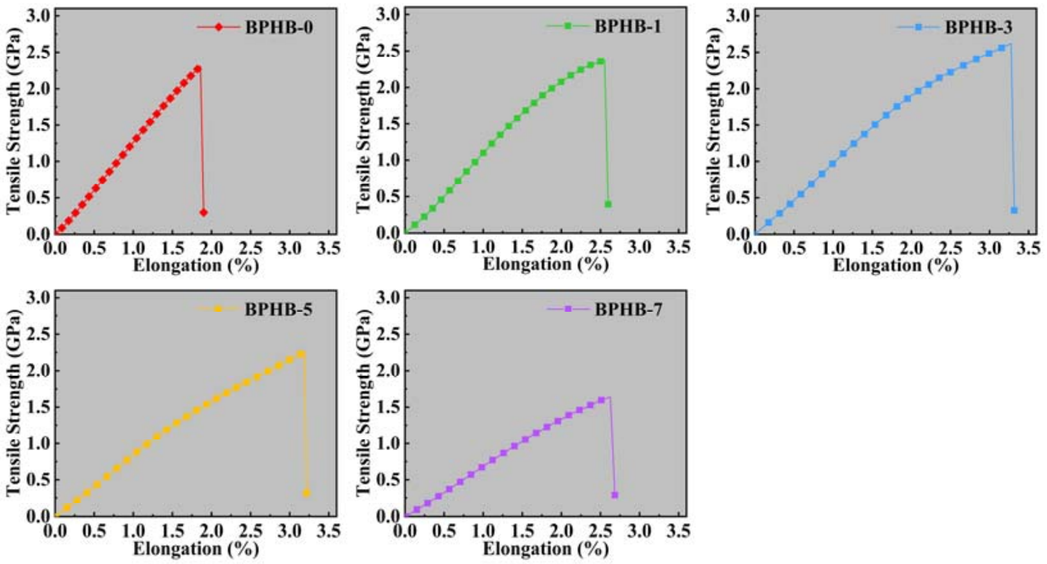

Figure S1. The typical tensile strength-elongation curves of the PI fibers with the various diamine molar ratios.

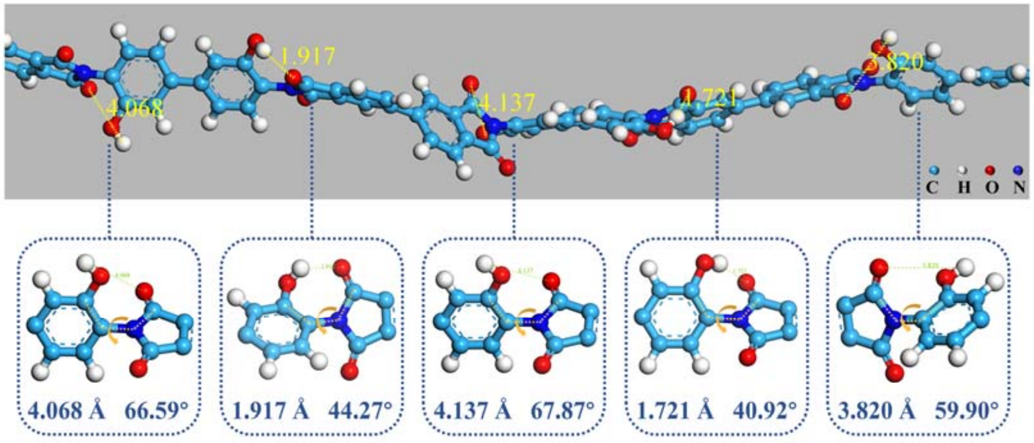

Figure S2. Simulated results of the dihedral angle between the imide ring and benzene ring containing -OH groups, as well as the atomic distance between the H atom of -OH and O atom of adjacent imide ring carbonyl for BPHB/HAB polymer chain in the lowest energy conformation.

Citation: Du, J.; Pu, C.; Sun, X.; Wang, Q.; Niu, H.; Wu, D. Preparation and Interfacial Properties of Hydroxyl-Containing Polyimide Fibers. *Polymers* **2023**, *15*, 1032. https://doi.org/10.3390/polym15041032

Academic Editor: Bożena Jarząbek

Received: 21 December 2022
Revised: 23 January 2023
Accepted: 26 January 2023
Published: 19 February 2023

Copyright: © 2023 by the authors. Licensee MDPI, Basel, Switzerland. This article is an open access article distributed under the terms and conditions of the Creative Commons Attribution (CC BY) license (https://creativecommons.org/licenses/by/4.0/).

The conformation of the BPDA/HAB polymer chain at the lowest energy is simulated using Materials Studio 8.0 software in Figure S2. Under the geometrically optimized conformation, the dihedral angles between the imide ring and benzene ring containing -OH groups are greater than 40°, implying that HAB moieties present a non-coplanar conformation with the backbones. In this situation, the probability of the distance between H atom of -OH and O atom of adjacent imide ring carbonyl being less than 2.6 Å is merely 40%. Generally, coplanar conformation along with atomic distance ($d_{(H...O)}$ < 2.6 Å) are considered to be positive factors for the formation of intramolecular hydrogen bonds [39,44,45]. Consequently, the tendency to form intermolecular hydrogen bonds (-OH···O=C-) in BPHB fibers is simultaneously enhanced when more HAB monomer is introduced into BPDA/*p*-PDA/BIA molecular chains.

References [39,44,45] are cited in the main text.

Table S1. The surface elemental compositions of BPHB fibers with various diamine molar ratios.

**PI Fibers**	**Element Content (%)**			**O/C**
	**C 1s**	**N 1s**	**O 1s**	
BPHB-0	80.62	3.63	15.75	0.19
BPHB-1	80.22	3.51	16.27	0.20
BPHB-3	77.38	3.84	18.78	0.24
BPHB-5	75.95	3.95	20.10	0.26
BPHB-7	74.13	3.80	22.07	0.30
